# Finally Throwing Those Wellies Away? Collaborating in Search of a Solution for Venice Flooding

**DOI:** 10.1007/s00267-022-01727-3

**Published:** 2022-10-08

**Authors:** Daniela Cristofoli, Benedetta Trivellato, Marta Micacchi, Giovanni Valotti

**Affiliations:** 1 Sda Bocconi School of Management, Milan, Italy; 2 Department of Sociology and Social Research, University of Milano-Bicocca, Milan, Italy; 3 Bocconi University, Milan, Italy

**Keywords:** Collaborative environmental governance, Collaborative inertia, Leadership, Orchestrator, Collaborative governance case database

## Abstract

Collaborative governance is often advocated as a way to address ‘messy’ problems that individual stakeholders cannot solve alone. However, whereas stakeholders’ participation brings a broad range of response options to public decision-making, the complexities of the perspectives at stake may also lead to conflicts and stalemates. This is especially true in collaborative environmental governance, where conflict is common and stakeholders’ interdependence in more than one arena tends to be frequent. Based on a longitudinal field study, we explore how to break stalemates in collaborative environmental governance when they occur, and move the collaboration towards a shared decision. The successful collaborative decision-making for the defence of Venice against floods represents our empirical setting. Our findings show that, in this context, the combined effect of three factors seems to be important to break stalemates and lead stakeholders towards a shared decision in collaborative environmental governance: stakeholders’ reactivation, fear of marginalization and leaders acting as orchestrators.

## Introduction

Governments are called to tackle wicked problems, demanding the pooling of resources and ideas among different actors in the pursuit of joint solutions, including to environmental challenges. The complexity and uncertainty of the problems addressed in environmental management require multiple actors to collaborate in collective decision-making and problem-solving (Folke et al. 2002, 2005; Armitage et al. 2009; Butler et al. 2015; Imperial et al. 2016). Collaborative governance does, in fact, bring together a variety of actors (i.e., individuals, agencies, and institutions) able to provide a broader range of response options that facilitates democratic natural resource management under changing conditions (Ostrom 2005). If, on one hand, stakeholders’ participation in public decision-making seems to be a basic component of successful environmental governance, on the other hand, the complexities of the perspectives they bring into the decision-making process may also lead to conflicts and stalemates (Baird et al. 2019). Stakeholders may agree that a solution needs to be found, but may remain trapped in a never ending dance, unable to come to a shared decision (Baird et al. 2019; Imperial et al. 2016).

The aim of our paper is, therefore, to explore how to break stalemates when they occur in collaborative environmental governance, and move the collaboration towards a shared decision. In particular, based on Ulibarri et al. (2020)’s dynamic framework and drawing from insights provided by previous use of the Collaborative Governance Case Database (Douglas et al. 2020), we aim to explore which factors may impact on stakeholders’ interaction in collaborative environmental governance, thus hindering or promoting their convergence towards a shared decision.

For this purpose, we adopted a longitudinal perspective (Imperial et al. 2016; Ulibarri et al. 2020) and conducted a case study analysis (Eisenhardt 1989; Eisenhardt and Graebner 2007). We analyzed the evolution of the stakeholders’ interaction over time in a successful case of collaborative environmental governance, where stalemate occurred and was broken, thus leading stakeholders to converge towards a shared decision. The collaborative governance process concerning the protection of Venice from “high flood water” represents the empirical setting for our study.

In 1966 Venice was inundated by a dramatic flood, making clear that a solution had to be found to “save Venice”, and that collaboration among multiple actors was necessary. In 1984 the Italian Government opened the decision-making process to public stakeholders with specific competences and expertise to address the problem, and created an institutionalized arena to facilitate their interaction. Other actors - such as businesses, environmentalist groups and associations, hoteliers and shop owners’ associations, and Venice citizens more generally - were engaged in a broader less institutionalized arena through consultation and information strategies. After years of studies, in 2000 the so-called “Mose” project emerged as a possible way to defend Venice from the “high floods”: it consisted of a system of mobile barriers laying on the bed of the lagoon. When a tide over 110 centimetres was expected, the mobile barriers would come to the surface and block the three canals joining the lagoon to the sea, thereby closing the high tide outside the lagoon. Three years later, the project still lacked a decision that would lead to its implementation. Contrasting positions led to a stalemate and made the collaborative governance “dormant” for years. In the end, however, the stakeholders converged toward a shared solution, and in 2003 the project was finally launched. For the purposes of our study, we then focused our analysis on the 2000–2003 time period. We chose to explore this successful case, as extreme cases are to be preferred when the aim is to explore a specific manifestation of a phenomenon (Patton 1990).

The results shed light on the combination of three factors which, in this context, contributed to break the stalemates and move the stakeholders towards a shared decision: stakeholders’ reactivation (linked to new energies engaged in the collaboration), fear of marginalization (due to social control mechanisms exerting pressure on participants), and leaders acting as orchestrators between the positions of the collaboration’s participants and those of the stakeholders they represent. Secondly, our results shed light on the importance of the individual and “micro-level” positions to better understand collaborative governance dynamics: as collaboration is first and foremost among individuals, their values, motivations, personality, emotions, and so on do matter in influencing collaboration dynamics and results. Thirdly, our results contribute to fill a gap in the collaborative governance literature, as few studies investigate which factors may affect collaborative governance performance over long timespans (Emerson and Nabatchi, 2015).

The article proceeds as follow: we will first lay out the conceptual foundations of the study, defining collaborative environmental governance, and review the extant literature about collaborative governance in the environmental field. Advantages and drawbacks of stakeholder participation in public environmental decision-making are thus highlighted. Secondly, we will describe the empirical setting of our study and tell the story of the Venice defence as a successful case of collaborative environmental governance. The third section presents the study method. Then, we will display and discuss the results. Some conclusions for further research on collaborative environmental governance are provided in the last section of the paper.

## Collaborative Governance in Environmental Management: Concepts and Theoretical Background

We focus on stalemates as particular events that may occur in collaborative environmental governance processes, and explore how to break them and move collaboration towards a shared decision. Drawing on the Collaborative Governance Case Database (CGCD) (Douglas et al. 2020), we define collaborative environmental governance as “a collective decision-making process based on more or less institutionalized interactions between two or more actors that aims to establish common ground for joint problem solving and value creation” (Douglas et al. 2020, p. 498). As indicated in the CGCD, collective decision-making processes may involve only government entities, only non-government entities, or a mix of the two (Douglas et al. 2020). We will therefore use “collaborative environmental governance” to indicate participatory public decision-making processes addressing environmental problems that may involve only government entities, or which may also include private actors, non-profit organizations and citizens in more or less institutionalized arenas for interaction.

### Advantages and Drawbacks of Collaborative Environmental Governance

There is ample evidence that collaborative environmental governance can both enhance the environmental standard of the outputs of decision-making processes and improve the implementation of these outputs (e.g., Biddle and Koontz 2014; Biddle 2017; Newig and Fritsch 2009; Scott 2015; Jager et al. 2020).

Collaborative governance eases creative problem-solving as a result of the combination of stakeholders’ capabilities and resources (Ansell and Gash 2012), and, via the engagement of both public and private actors in consensus-oriented decision-making, it makes environmental management more democratic (Mostert et al. 2007; Stringer et al. 2006). Stakeholders’ participation also allows to share multiple views and diverging interests, thus promoting more thoughtful and innovative decisions (Beierle and Cayford 2002; Sirianni 2009; Margerum 2011; Trivellato et al. 2021). Collaboration allows to share expertise, knowledge, procedural arrangements and other capacities, which are important to generate innovation (Torfing et al. 2020; Mariani et al. 2022). Moreover, decisions resulting from participatory processes reduce the risk of noncompliance and opposition, thus facilitating implementation (Bulkeley and Mol 2003; Innes and Booher 1999; Cristofoli et al. 2022).

On the other hand, stakeholders’ participation in public environmental decision-making can lead to open conflicts, which may result from individuals framing the problem differently because of differences in knowledge, beliefs, and values (Adams et al. 2003; Young et al. 2010). Finding a common ground towards collective action can be difficult (Butler et al. 2015); developing a shared vision is not easy (Shindler et al. 2002; Cheng and Mattor 2006; McCaffrey et al. 2013; Urgenson et al. 2017); and reaching a “zone of agreement” might be particularly challenging in environmentally sensitive and culturally dense areas (Collins et al. 2010; Franklin et al. 2014). Urgenson et al. (2017) identify ten main challenges to develop a shared vision or, borrowing Emerson et al.’s words, “a shared theory of action” (2012: 11). Stakeholders’ inability to move from agreement on broad ideals to specific recommendations combines with the “threat of litigation”, as powerful interest groups decline to participate in the consensus-building process on ideological grounds, delaying decisions by analysis or appeal. Moreover, stakeholders’ diverging values might come into conflict, ending up in a disproportion between the socio-economic frame and the environmental one. As the former appears to be less powerful than the latter, the collaborative process might come to a standstill. In this perspective, Bodin et al. (2020) remark that stakeholders with different interests in a common resource often form coalitions that cause competing positions, and that this may lead the decision-making process to a stalemate. Differences in interests, beliefs, or worldviews result in differences in problem frames, as cognitive interpretations that give meaning to complex environmental phenomena; this plurality of ideas and views often leads to controversy and stalemate (Runhaar and van Nieuwaal 2010). This is especially true in collaborative environmental governance, where issues feature high conflictual situations and it is difficult to reach an agreement among multiple stakeholders’ perspectives (Baird et al. 2019).

Based on these considerations, this paper aims to identify the factors that are able to hinder and foster stakeholders’ converge towards a shared decision. The successful case of the collaborative governance process dealing with Venice defense from “high flood water” will represent the empirical setting for our study.

## The Evolving Problem of Protecting Venice

Venice is a system of islands at the centre of a large lagoon, connected to the sea by three small canals. Every six hours, the tide enters and leaves the lagoon through these canals, ensuring that water is exchanged between the lagoon and the sea.

On November 4, 1966, an extensive flood inundated Venice and the surrounding villages. The seawater reached a depth of 1 meter and 94 centimetres in Saint Mark’s Square, putting the city at risk and causing untold inconvenience to all the residents of Venice. “*I felt like the Adriatic Sea wanted to fill my home*” recalls one resident of Venice.

After this event, Venice has been inundated by the Adriatic Sea several times a year and its survival is increasingly at risk. “*Save Venice from the high tide*” has thus become a goal that residents, together with institutional and non-institutional actors, aim to achieve. However, none among those actors on its own possesses all the resources and expertise needed to face the problem of protecting Venice: collaboration among multiple stakeholders was the only possible way out.

The Italian Parliament, first, transferred the decision-making power regarding the protection of Venice from the local to the national Government in 1973, as the latter was in a position to mobilize additional financial, technical, and collaborative resources. The Italian Government acted through its Ministries, the Ministry for Public Works *in primis*, and its local level spin-off, i.e., the Venice Water Authority. Then, in 1984, the Italian Government took two important decisions. On one hand, it decided to give to a single body the responsibility for all the actions related to the protection of Venice, founding the Venezia Nuova Consortium (CVN) to oversee all the relevant public works. The CVN consisted of more than twenty leading construction companies at the national and local level. On the other hand, the Italian Government set up a roundtable as the *locus* where all the key actors interested in protecting Venice could pool their resources and skills and make the best decisions: the Committee for Policy, Coordination, and Control (called the ‘*Comitatone’*). The *Comitatone* was responsible for directing, coordinating, and controlling all the initiatives taken to protect Venice. It convened the stakeholders who had institutional responsibility for the solution of the high tide issue and had the expertise and skills to tackle it: the Italian Prime Minister and his Ministers (the Ministries of the Environment, Public Works, Cultural Heritage, and Scientific Research), the Venice Water Authority, the Venice local government, the Veneto’s regional government, other municipalities located in the lagoon. To facilitate stakeholder interaction, a set of coordination rules was also established, and a third-party role was identified: the *Comitatone* decided by consensus of all its members (who had the same decision-making power) and was chaired by the Italian Prime Minister (who also called its meeting, set the agenda and tried to mediate between the contrasting positions of its members).

In 1989 the CVN completed the conceptual design of the “Mose project”, consisting of mobile barriers to be installed on the bed of the lagoon underneath the three canals joining it to the sea. When the tide is normal, the mobile barriers are designed to lie on the bed of the lagoon: this safeguards the exchange of water in the lagoon and avoids damaging the port and fishing industry. When a tide over 110 centimetres is expected, the mobile barriers are designed to come to the surface, blocking the three canals and closing the high tide outside the lagoon.

The project was examined in 1992 by the Venice Water Authority and by a committee of experts from leading international engineering companies: they agreed that it should go ahead, and the Veneto regional government supported them. Nevertheless, Venice’s local government was not convinced of the benefits of the mobile barriers, and asked the *Comitatone* to submit the project to the Ministries of the Environment and Cultural Heritage for an Assessment of its Environmental Impact (EIA).

In December 1998, the Ministry for the Environment and the Ministry for Cultural Heritage submitted their negative EIA in relation to the mobile barriers: they claimed the project could hurt the survival of the lagoon, and pointed out that other (less expensive and easier) solutions were available.

To tackle the conflicting opinions among its members, the *Comitatone* asked the Venice Water Authority to examine the pros and cons of the mobile barriers once again. The *Comitatone* was to take its decision based on the results of these studies by the end of the year 2000: either embarking upon the final design of the project, or discarding it altogether and search for other solutions.

The December 2000 deadline passed without a decision, and the collaborative governance process remained dormant among contrasting positions. Only on April 3, 2003, the *Comitatone* gave the go-ahead for its implementation and the foundation stone was laid on May 3, 2003. In the following we will focus our analysis on the period 2000–2003, during which the stalemate was broken and stakeholders’ interaction evolved until a final and shared decision was taken.

## Method

Between January 2000 and April 2003, the stakeholders involved in the *Comitatone* sought a shared decision for four times. Twice they unanimously agreed to postpone the final decision, thus remaining in a stalemate; twice, instead, they came to a final decision and took a step forward in the direction of the problem’s solution. The stalemate was definitively broken in April 2003, and the mobile barriers project was started in May 2003.

Thus, we focused our analysis on the 2000–2003 timespan, and divided the collaborative governance process into four phases (according to the four attempts at reaching a shared decision).

We took a comparative and developmental perspective (Imperial et al. 2016; Ulibarri et al. 2020) and drew on a longitudinal case study to investigate the collaborative governance process. We considered each phase as an autonomous case, and conducted both a comparative and a longitudinal analysis. Comparative analysis is useful as a basis of replication (Yin 1984; Eisenhard 1989): it helped us understand what may work or not in collaborative governance. Longitudinal analysis enables the observation of interactive temporal patterns and contextual influences (Pettigrew 1990): it helped us understand how stakeholders’ interaction evolved over time, thus moving the collaboration from stalemate to a shared decision.

### Data Collection

We collected data dealing with the actions carried out by the stakeholders interested in protecting Venice between January 2000 and June 2003. For this purpose, we drew on two different data sources: newspapers analysis and stakeholder documents.

First, we collected and analysed all the articles dealing with the protection of Venice published by two local and one national newspaper (4502 articles). Local newspapers published about one article a day discussing the protection of Venice and took different positions about the implementation of the mobile barriers: one of the local newspapers we analysed was in favour, the other was against the Mose project. The national newspaper dedicated a great deal of attention to the implementation of the mobile barriers, and reflected mostly the national government’s views. Second, we collected and analysed 338 documents (i.e., press releases, agendas, minutes, and resolutions from meetings, private notes, and correspondence, newsletters, brochures, flyers, etc.) produced by all the actors involved in the safeguard of Venice (i.e., Venice’s local government, the regional government, the Venice Water Authority, the CVN, the Italian Government, the Public Works Ministry, the Environment Ministry, the *Comitatone*, environmentalist groups, Venice Hotelier and Shop Owner Associations, Consumer Associations, etc.).

### Data Analysis

Data were transformed in accordance to the three phases of data storing, managing and processing (Miles and Huberman 1984).

The first phase was descriptive and aimed to reduce data into a set of events, listed in chronological order, occurring during the collaborative governance process. It relied on a coding process. The database was searched for critical moments and events relating to the actions carried out by relevant stakeholders. The search was conducted independently by two researchers.

The second phase was analytical and aimed to build categories of concepts (Locke 2001). Articles and documents were coded by the researchers so as to lead empirical data to analytical categories emerging from the literature (Ulibarri et al. 2020). Based on Ulibarri et al. (2020), and drawing from previous use of the Collaborative Governance Case Database (Douglas et al. 2020) three categories emerged from the analysis. They can be related to: stakeholders’ participation, which identifies and lists all the stakeholders participating in the public decision-making and their position towards the Mose’s implementation; collaborative governance mechanisms, which refer to the whole of mechanisms employed to make stakeholders converge towards a shared decision; and leadership, which relates to the leaders’ tasks in framing the agenda of the collaboration, sustaining deliberation, managing power imbalances, and promoting the achievement of common goals.

The third phase of the data transformation process was interpretative and searched for a relationship among the categories of concepts we identified. We started to process data once concepts were categorized: we tried to understand how the various categories of concepts could be linked into a coherent framework. In particular, we looked for an explanation of the relationship between stakeholders’ participation in the collaborative decision-making process and the final decision, thus shedding light on which factors may hinder or promote their convergence towards a shared decision.

We used the triangulation of sources to ensure the intercoder reliability of the qualitative analysis (Denzin 1978; Strauss and Corbin 1990; Denzin and Lincoln 1994). We then used researcher triangulation to curb the effects of the researchers’ perceptions by arranging meetings to discuss data analysis with colleagues. Also, we tried to look for data supporting other explanations, and searched for the best match between data and interpretation. To minimize the problems of recollection, we also developed extensive triangulation during data collection and analysis as we confronted data and interpretation across documents.

## Findings

In order to better understand the evolution of the relevant events, we have divided the collaborative decision-making processes into four phases, which took place during the years 2000–2003. In this section, we describe what happened during each of the four phases, so as to highlight the dynamics and mechanisms that will be the basis for the discussion in the next section.

### Phase 1. January 2000 – July 2000

The *Comitatone* was expected to decide what to do about the mobile barrier project based on the studies by the Venice Water Authority: either to proceed with its implementation or definitively abandon it and look for another solution. However, several things changed in the political scenario in just a few months.

Venice was administered by a Mayor who was born locally and represented the spirit and aims of Venice. In the event of high tides, the Mayor asked residents to “*put their Wellingtons on*”. In January 2000 he decided to resign in order to run for the Presidency of the regional government. A local election campaign, then, started: the new Mayor was expected to be elected in April 2000. Two opposing coalitions emerged, adopting a different position with regard to the problem of protecting Venice. The left-wing coalition, including left-wing and environmentalist parties, was traditionally against the mobile barriers because they would hurt the long-term survival of the lagoon, whereas the right-wing coalition was in favour. However, the candidate for the left-wing coalition was the former Minister for Public Works, one of the advocates of the mobile barriers. As a candidate in Venice’s local election, he invited the people of Venice “*to take their Wellingtons off*”. Due to the local electoral campaign, the Italian Prime Minister decided to postpone the meeting of the *Comitatone*, in order not to interfere with the local election results, both at the municipal and regional level.

The candidate of the left-wing coalition was elected as Mayor of Venice in April 2000. At the regional level, the previous right-wing President was re-elected. Meanwhile, the Italian Prime Minister was forced to resign. Another Prime Minister was appointed by the left-wing and environmentalist coalition, and new Ministers were appointed. Just after his appointment, the new Minister for Public Works stated that the protection of Venice would be a priority during his term of office and guaranteed that the *Comitatone* would convene shortly. The meeting of the *Comitatone* had been awaited for over a year, since March 1999, when the Ministries of the Environment and Cultural Heritage submitted their negative EIA in relation to the mobile barriers, and the Italian Prime Minister was then forced to postpone the decision.

While waiting for the *Comitatone* to meet, two contrasting coalitions emerged. On one hand, the Ministries of the Environment and Cultural Heritage opposed the mobile barriers, claiming they would not solve the problem of protecting Venice, and proposing other solutions. The Venice City Council also shared this position, as embodied by its left-wing and environmentalist majority. On the other hand, the Venice Water Authority, the CVN and the Ministry for Public Works, supported by Veneto’s regional government, promoted the implementation of the mobile barriers as the only chance to save Venice from the high tides.

The Italian Prime Minister adopted a position that was midway between these two coalitions. He reaffirmed that the project would be carried forward in compliance with the EIA requirements, and with the conditions set by the *Comitatone*.

The Mayor of Venice also took an unusual stance. He was representing the Venice City Council, whose majority was against the mobile barriers, and acted on behalf of it within the *Comitatone*. However, he had previously been Minister for Public Works and had supported the implementation of the barriers during the Nineties. Consequently, he took a stance midway between the City Council majority and the opposition.

Whilst waiting for the *Comitatone* to meet, environmentalist groups attacked the prototype of the barriers: they hired a boat and staged a public demonstration against their implementation.

Before the meeting of the *Comitatone*, the Venice City Council agreed upon the position that the Mayor of Venice would uphold during the meeting: the Council agreed with the implementation of public works that had already been approved, whereas any additional public works (including the mobile barriers) should be postponed.

The *Comitatone* meeting finally took place on July 12, 2000. The Minister for the Environment resisted the pressure put on him by all the other stakeholders by saying “*No*” to the mobile barriers, he cut relationships with the *Comitatone* members, and the Italian Prime Minister was forced to postpone the decision. The *Comitatone* agreed that the final decision would be taken by the Prime Minister by December 2000, with the implication that the collaborative governance process remained in a stalemate.

### Phase 2. August 2000 – March 2001

The Fall of the year 2000 saw Saint Mark’s Square inundated with sea water as it did every year. Saint Mark’s Square is submerged when the tide water reaches 84 centimetres: in November 2000, it reached 144 centimetres, prompting the President of the regional government to call for the mobile barrier project to go ahead. Likewise, the Mayor of Venice claimed for an immediate solution to Venice’s problems.

The high tide continued to submerge Venice several times a month, but in January 2001 the Italian Parliament decided to reduce the funds allocated to the protection of Venice as a result of the strain on Italian public funds. Since 1966, the Italian Parliament had allocated part of its annual budget to Venice’s local government, its regional government and other local governments around the lagoon in order to fund the work needed to defend Venice. Cutting governmental funds was putting Venice’s survival at risk. Thus, economic dynamics also got in the way of a solution. Still, environmentalist groups said there was no cause for panic because of the floods, and that solutions other than the mobile barriers should be explored. On the back of these claims, environmentalist parties reinstated their position against the barriers.

Members of the *Comitatone* continued to depend on each other for a solution. Although the Ministry for Public Works continued to demand the implementation of the barriers, the Ministry for the Environment continued to oppose it, thus forcing the Prime Minister to take a decision.

In September 2000, environmentalist groups arranged a demonstration against the barriers: a long queue of boats paraded through the lagoon to raise awareness of the risks related to the project. After floods occurred in November, shop owners and hoteliers organised a “Wellingtons day” by closing shops and restaurants for a day in protest against the incapacity of the authorities to come to a decision. Residents also stressed the need to do something: a poll showed that 56% of them were in favour of the mobile barriers.

In the end, members of the *Comitatone* continued to act within its norms and procedures, even if a formal meeting was not convened. The Minister for Public Works demanded a decision, whereas the Minister for the Environment supported the idea of exploring other solutions for Venice’s problems. Thus, the Prime Minister once again had to mediate between the contrasting positions of the *Comitatone* members.

In order to mediate between the Minister for Public Works and the environmentalist parties, the Italian Prime Minister took another partial decision. On March 15, 2001, the Italian Government decided to go ahead with the mobile barriers, but it also stated that further studies were needed before the project became fully operational. It did not establish a deadline for the completion of these studies; as a result, the implementation of the barriers was postponed *sine die* and the collaborative governance process remained in a stalemate.

### Phase 3. April 2001 – December 2001

In the Spring of 2001, a new right-wing coalition won the national elections, and the new Prime Minister appointed new Ministers. As soon as the new Minister for Public Works was appointed, he immediately announced that he wanted to proceed with the implementation of the mobile barriers. In September 2001, the Italian Government declared that new funds were to be allocated to implement public works, but the Parliament reduced the amount in November 2001 because of the critical situation of the Italian economy.

Further to the declaration by the Minister for Public Works, the President of Veneto’s regional government wrote to the Italian Prime Minister and asked him to convene the *Comitatone*. He was confident that a final decision would be needed soon, but the *Comitatone* had to meet for it to be taken.

While waiting for the results of the studies requested by the Prime Minister, the usual two coalitions emerged, trying to influence the results of the problem-solving process. On one hand, the President of Veneto’s regional Government announced that the *Comitatone* was to meet by December. On the other hand, the Venice City Council approved a deliberation concerning the protection of Venice but without taking a position about the implementation of the mobile barriers.

At 17.07 pm on December 7, 2001, the *Comitatone* agreed to give the CVN the task of preparing the working plan for the mobile barriers. The Mayor of Venice pointed out that the barriers would be ineffective if a number of integrated actions to protect Venice from the high tide were not implemented. All the other *Comitatone* members agreed that it was necessary to implement these actions together with the barriers. In the end, the *Comitatone* unanimously decided to go ahead with the final design of the mobile barriers.

A decision was finally taken by the *Comitatone:* its members unanimously decided to go ahead with the next step of the project, and asked the CVN to prepare the working plan. A decision was successfully taken and the stalemate was broken.

### Phase 4. January 2002 – May 2003

In autumn 2002 the floods once again submerged Venice. In December, the Minister for Public Works agreed on a list of public works that the Government intended to launch as soon as possible, including the mobile barrier project. Consequently, the Italian Prime Minister decided to allocate new funds to public works, but the Minister for the Economy did not concede because of the dire state of the Italian economy.

In March 2003 the *Comitatone* had to meet to decide on the implementation of the mobile barrier project. Venice’s local government had to state its position, which proved difficult because of conflicting views within the City Council: the opposition parties agreed with the project, whereas the majority party was against it. Because of this inability to find a common stance within Venice’s local government, the *Comitatone*’s meeting was postponed three times. Had the *Comitatone* decided to take this decision without the agreement of Venice’s local government, the relationship between the Italian Government and Venice’s local government would have crumbled.

In June 2002, environmentalist groups arranged a public hearing to explain to residents why they were opposing the mobile barriers. Other public meetings were organized in the following months. Meanwhile, the Association of Architects set aside the implementation of the barriers, and pressure groups proposed a referendum asking local residents to express their opinion about the barriers.

As these public manifestations against the barriers were unfolding, a *Comitatone* meeting was expected. The Venice City Council tried to agree upon the position that the Mayor of Venice was to take during the *Comitatone* meeting. Despite repeated difficulties, the Council ultimately approved a document containing 11 points for the implementation of a number of public works to protect Venice, before starting on the implementation of the mobile barriers.

The *Comitatone* met on April 3, and the Mayor stated the position of the Venice City Council. The Italian Prime Minister accepted all the demands made by the City Council, and all the other *Comitatone* members agreed. Thus, a final decision was taken by the *Comitatone* with regard to the mobile barriers by way of consensus of all its members. The stalemate was finally broken, and the foundation stone was laid by the Italian Prime Minister on May 3, 2003.

## Discussion

As Baird et al. (2019: 16) argue, “collaboration is a common rallying cry when making decisions about environmental and natural resources”. On one hand, environmental issues’ complexity requires collaboration among multiple actors bringing diverse resources and competences. On the other hand, environmental issues feature high conflictual situations where it is difficult to reach an agreement among multiple perspectives (Baird et al. 2019), thus making the collaboration dormant and leading to stalemates (Imperial et al. 2016). The extant literature sheds light on the importance of stakeholders’ convergence for collaborative governance success (Ansell and Gash 2008; Emerson et al. 2012; Emerson and Nabatchi 2015), but “important questions remain as to the specific mechanisms that drive these relations” (Jager et al. (2020), pag. 384; Bodin 2017; Emerson et al. 2012; Scott 2015).

The results of our study shed light on certain factors that impacted on stakeholders’ interaction, hindering or promoting their convergence towards a common solution. In this way, they highlight how stalemates were broken when they occurred in this case of collaborative environmental governance, thereby moving the collaboration towards a shared decision.

Firstly, by adopting a comparative and longitudinal perspective, coherently with the extant literature, our study shows how the misalignment of stakeholders’ positions seems to hinder their convergence towards a shared decision, thus generating a situation where stakeholders’ are trapped in a never ending dance, unable to take the final decision.

In the first phase, stakeholders acknowledged that a solution to the high tide problem required a joint effort and did not refrain from direct confrontation. However, there was a clear misalignment of interests and values, with two groups of actors strongly affirming their position in favour (among them the Venice Water Authority, the Ministry for Public Works, the CVN, and the Veneto regional government) or against (the Venice City Council, the Ministry for the Environment) the implementation of the mobile barriers. This phase was therefore marked by a continued inability of the *Comitatone* members to reconcile their perspectives. Their behaviours either hindered the final decision (by postponing the *Comitatone* meeting), or stressed its urgency (by pointing out the need to convene the *Comitatone* as soon as possible). Ultimately, the Italian Prime Minister took upon himself a leadership role and postponed the decision, with the *Comitatone* agreeing that a decision would be taken by the Prime Minister himself before December 2000.

In phase 2, the process evolved in a way similar to phase 1, though particularly high tides and the resulting floods in Autumn 2000 made actors more vocal both in favour and against the mobile barriers. Those who were against, such as the Venice City Council and the Ministry for the Environment, argued that the mobile barriers would over time hurt the lagoon, and that other solutions had to be explored. Actors continued to be aware of their interdependencies, but the misalignment of the positions expressed by the two opposing coalitions could not be overcome. In the end, the Prime Minister decided in favour of the go ahead, but with the requirement that additional studies be completed first, with no firm deadline for such completion.

While the first two phases were characterised by a substantial stalemate due to the misalignment of stakeholders’ positions, the analysis of the third and fourth phases sheds light on the factors that were able to break the stalemate and move the collaboration towards a shared decision.

In the third phase, the collaborative governance effort began to mark a shift. With a new Prime Minister and new Ministers overseeing Public Works, Cultural Heritage, Environment and Scientific Research, new actors with new resources and energies entered the *Comitatone*. Within the *Comitatone*, the only major stakeholder still against the mobile barriers was the Venice City Council, whose Mayor, however, was personally in favour of the project. The misalignment of positions, while still present, therefore began to shrink. This declining misalignment, in turn, started to act as a social control mechanism exerting pressure on the Venice City Council within the *Comitatone*. Stakeholders’ views started to converge in the direction of a possible joint solution that could finally break the stalemate. Leadership dynamics also marked a shift in this phase relative to the two previous phases: the new Prime Minister pushed for the *Comitatone* to finally meet. In this situation, the Mayor of Venice felt the pressure from the *Comitatone* members, but still could not disregard the position of the Venice City Council he was actually representing. A meeting of the *Comitatone* in December 2001 at last succeeded in favouring a convergence and breaking the stalemate, with members agreeing to go ahead and ask the CVN to prepare a working plan.

The fourth phase showed a further evolution towards the breaking of the stalemate and stakeholders’ convergence. Facing the social pressure from members of the *Comitatone* who were overwhelmingly in favour of the mobile barriers, the Mayor was able to obtain from the City Council the mandate to agree to a go ahead within the *Comitatone* itself, as long as a number of other measures related to public works were concurrently agreed upon. Once these compromises were set and accepted, the committee could finally and unanimously decide in favour of the mobile barriers. Social pressure was then able to lead towards a compromise solution and the project was finally set on its way to be implemented. The Mayor of Venice pushed for the compromise and agreed to a decision he had always supported personally, but which was not supported by the institution (the Venice City Council) he was representing within the *Comitatone*. The Mayor therefore contributed a critical leadership function in this last phase, which led the Prime Minister to finally lay the foundation stone for the project in May 2003.

Our study therefore suggests that a combination of three factors has been able to break the stalemate and lead stakeholders to converge towards a shared decision: stakeholders’ reactivation, as new energies enter the collaboration; fear of marginalization, as a social control mechanism exerting pressure on the collaboration’s participants; and leaders as orchestrators who are able to blend different positions both internal and external to the collaboration.

In the following, we will first reflect on the impact of each of these three factor on stakeholders’ interaction, thus showing its ability to promote stakeholders’ convergence. Secondly, we will show how it is their combination that, in our case, allows to break the stalemate and move the collaboration towards a shared decision.

### Stakeholders’ Reactivation

As Imperial et al. (2016) and Ulibarri et al. (2020) show, participants may change during the collaboration’s evolution. Ulibarri et al. (2020) argue that stakeholders can experience “burnout” due to all the energies, efforts and commitment dedicated to the collaboration and, once stability is reached, they may decide to leave. Moreover, people change jobs, get promoted, or retire, and this creates a necessary turnover in participation. This can be a cause of collaborative governance decline, but new participants may also have new and different values, motives, aims and this can lead to a reorientation or recreation of the collaborative governance. This reactivation of the collaboration may therefore be linked not only to new individuals entering the interaction processes, but also to new dynamics and ideas that reinvigorate existing participants. New resources and energies, then, may allow a dormant collaboration to reawaken. That is what happened in our case, where the turnover of the participants joining the *Comitatone* is the starting point of a new process where conflict is not prevalent anymore, and an alignment among different positions starts to become possible.

### Fear of Marginalization

A social control mechanism is a second factor which seems to play a role in favouring stakeholder convergence. Jager et al. (2020) investigate the importance of mechanisms such as social learning, trust building, shared norms, mutual gains and conflict resolution to mediate the relationship between stakeholders’ participation and environmental effectiveness. Our work adds to this study, by shedding light on “fear of marginalization” as an additional important mechanism able to favor stakeholders’ convergence. In fact, Häge (2013) employs fear of marginalization to describe actors’ strategic decisions to coordinate and collaborate with others in order to prevent that their positions be ignored. Similarly, Baird et al. (2019: 12) describe it as “the fear that non-participation in the coalition would create an inability to influence the decision-making process”. As stakeholders fear that their own interests will not be taken into consideration in the pursue of a common cause, this mechanism fosters the success of the coalition by inducing action. In this light, fear of marginalization establishes itself as a “valid reason for individuals to engage in natural resource management” (Baird et al. 2019: 13), supporting leaders in addressing ongoing conflict. Our case, in phases 3 and 4, shows the importance of such a mechanism. When all *Comitatone* members agree to proceed with the Mose (phases 3 and 4), the Venice City Council remains the only actor against the project: this eventually facilitates the search for a compromise, consisting in the acceptance of the Mose by the City Council, as long as additional works to safeguard the lagoon are put in place. The *Comitatone* accepts, and the final decision to start Mose’s implementation is taken.

### Leaders as Orchestrators

A specific character seems to have a potentially critical role in promoting the stakeholders’ convergence in collaborative environmental governance, according to our data. The collaborative governance literature has investigated the role and tasks of collaborative governance leadership (Crosby and Bryson 2005; Ansell and Gash 2008; Ansell and Gash 2012; Sørensen and Torfing 2012; Emerson and Nabatchi 2015; Crosby et al. 2017; Cristofoli et al. 2021), showing that leadership is a key factor for successful collaboration. Leadership is widely recognized to initiate, promote, and sustain collaboration in many ways; from catalysing the group to work together, to upholding the health and integrity of the collaborative process, to mediating conflicts between stakeholders by disclosing possible win–win gains, to crafting a solution when stakeholders lead the consensus-building process to a halt (Susskind and Cruikshank 1987; Ryan 2001; Ansell and Gash, 2012; Torfing 2016). Coping with participants’ reluctance and inaction in sharing power (Gray 1989; Ran and Qi 2019), leaders can move stakeholders from conflict to coalition, thereby ending a stalemate (Innes et al. 2007; Fullerton 2009). Besides different types of leaders, scholars showed that leadership changes over time not only with reference to how many actors start playing the leading role (Ulibarri et al. 2020), but also with respect to who becomes the leader. In this light, Fliervoet et al. (2016) highlighted the importance of the changes occurring in the general system context, pointing out that the shifting of the central actor may impact significantly on collaboration performance. In our case, leadership plays a role in all four phases, but it is only in phase 3 and 4, when a new figure emerges, that it favours stakeholders’ convergence. In all four phases we witness the Prime Minister’s leadership. He is the one who convenes the *Comitatone* meeting, mediates among stakeholders’ contrasting position and sets the defining steps at certain crucial moments. Nevertheless, this is not enough to lead the collaborative governance process out of the stalemate. A new character emerges who shares the leadership with the Prime Minister, even if with different tasks and on a different arena. The new character shares certain features with what is known as an orchestrator, that is, an actor who is able to combine different perspectives and positions within a single symphony. Bartelings et al. (2017: 355) define orchestrational work as “the role in which the orchestrator consciously integrates and therefore fine-tunes activities which have to be executed by network partners from various organizations to deliver concrete jointly arranged results”. In our case, the orchestrator acts across the boundaries between the *Comitatone* and the Venice City Council. He is the Mayor of Venice, who is able to combine the position of the *Comitatone’*s members with that of the City Council.

### The combined effect of the three factors

Last but not least, our results shed light on the combined effect of the above-mentioned insights to break stalemates and move the collaboration towards a shared decision. Stakeholder reactivation emerges in phase 1 and 3, but does not on its own allow to breake the stalemate and move the collaboration towards a shared decision. Similarly, fear of marginalization emerges in phase 2 and 3, but the final decision is not taken. Only in phase 4, when stakeholder reactivation combines with fear of marginalization and with the orchestrator’s action, the stalemate is definitively broken and the collaboration moves towards the final decision. In fact, it is only after new members and energies enter the *Comitatone*, when the social pressure mechanism (or fear of marginalization) leads the Venice City Council to try to reach a compromise, that the Mayor of Venice, in his role as orchestrator, facilitates stakeholders’ convergence, the breakthrough of the stalemate, and the formulation of a shared decision.

## Conclusion

Collaborative governance is often proposed as able to solve “messy” problems that individual stakeholders cannot solve alone. It allows to think out of the box and to create new and bold solutions (Sørensen and Torfing 2012; Ansell and Torfing 2014; Crosby et al. 2017; Hofstad and Torfing 2016). It is also considered able to break political stalemates by providing new perspectives and innovative win-win solutions (Torfing 2016). Nevertheless, collaboration is often a “difficult and painful process, featured by low processes to achieve negligible outputs” (Huxham and Vangen 2005: 7). This is especially true in environmental management, where “conflict is common, and bringing together stakeholders with diverse perspectives in situations of conflict is extremely difficult” (Baird et al. 2019, pag. 16). Paralysis of actions and decisions can be the undesired result of collaborative environmental governance, and stalemate can be its outcome.

Using a longitudinal case-study of an especially complex collaborative governance process, this paper shows how three factors may emerge as important to promote convergence: stakeholders’ reactivation over time, fear of marginalization, and leaders acting as orchestrators. Moreover, a combination of these three factors seems in this context to emerge as an effective path to break stalemates and move the collaboration towards a shared decision.

The results of our study therefore offer insights to both the literature on collaborative governance and that on environmental management.

Research on collaborative governance, typically, focuses on the conditions for successful collaboration, in the attempt to further develop Ansell and Gash’s model (2008). In particular, it focuses on dimensions such as starting conditions, institutional design, collaborative processes and facilitative leadership. Compliance with these conditions should facilitate the collaboration’s success and prevent it from entering a state of inertia. Our work investigates situations where stalemate occurs and, on one hand, confirms the results of the existing literature, while also on the other hand enriching them.

First, our study sheds light on the importance of adopting a micro-organizational perspective to further investigate collaborative governance. Collaborative governance is a form of collaboration that “brings together relevant and affected actors in networks and partnerships held together by a mutual recognition of the need to exchange and pool resources and constructively manage their different interests, ideas and perceptions in the pursuit of joint solutions to common problems” (Sørensen and Torfing 2021: 1591). However, organizations participate in the collaboration through their representatives. Collaborative governance is then, also, a collaboration among the individuals who participate in the meetings. This means that each individual participates with their own values, personality traits, cognitive biases, and emotions, and this can affect collaborative governance processes and outputs. This is particularly clear in our case, where the newly appointed Mayor of Venice represents the City Council that is against the mobile barriers, but he is also in favour of their implementation. It is this misalignment between a single individual’s position and the organization’s position that, finally, affects the collaborative process and contributes to reach a compromise solution.

Secondly, our study sheds light on the importance of adopting a network perspective to further investigate collaborative governance. Collaborative governance does not take place in a vacuum. Stakeholders invited to take part in a single collaborative process may also participate in other decision-making arenas. This creates a complex network of interdependence and reciprocal influences that can deeply affect outcomes. This is particularly clear in our case if we consider that the political actors sitting in the *Comitatone* are also involved in many other political arenas dealing, for example, with the national government’s stability.

Research on environmental management often requires collaboration among multiple stakeholders (Folke et al. 2002, 2005; Armitage et al. 2009; Butler et al. 2015). “Collaborative approaches which bring together a range of stakeholders in an iterative process of learning and doing are hypothesized to more effectively address system complexity, change, and uncertainty” (Baird et al. 2019: 16). However, collaboration can be difficult to manage and can lead to stalemate when environmental issues are addressed, given that these are complex issues that deal with different aspects of the political, economic, and social life. Therefore, stakeholders’ interdependence in more than one collaborative governance arena tends to be frequent. Our work highlights that these multiple interdependencies should be the focus of attention, possibly also during the institutional design phase, so that their potential impact is taken into consideration, and remedial mechanisms are put in place to the extent possible. In addition, the role of the orchestrator should also be taken into consideration as a possible figure who contributes to reconcile these multiple interdependencies and helps to reach compromises when interests alignment is out of reach. These considerations are closely linked to the topic of who should be included in collaborative governance arrangements, and add to Ansell et al. (2020)’s framework for distinguishing between the different motives and roles of both participants and non-participants. Our work suggest that attention should be given also to the fact that such motives and roles may change over time (for instance because of the changing political and/or institutional context, and the related changes in interdependencies), and that different motives and roles may co-exist at the same time within the individual participating in the collaboration (for instance because of differences between their own position and that of the organization they represent).

Lastly, our work also contributes to practice, as it pushes those who deal with the management of collaborative governance processes to pay specific attention to the three elements that emerge from our analysis. First, it encourages them to find ways to infuse new energy in the collaboration, which may, for instance, take the form of new participants, or new design and problem-solving methodologies. Second, our results serve as a reminder that social pressure may be a powerful tool to influence behaviours, and that searching for an acceptable compromise may help push that social pressure in a desirable direction. Third, our work suggests that more traditional leadership roles may be shared and/or enriched through reliance on an ‘orchestrator’ who may be able to blend different positions and help their convergence towards a common decision.

Being based on a single case study, our work does not aim at generalization, but rather to point at certain dimensions which may have been overlooked by the extant literature—such as the fact that individuals may concurrently participate in more than one collaborative governance process—and especially to highlight that such dimensions evolve over time. This implies that relevant dynamics and roles within the collaboration also change over time, with both positive and negative outcomes. This study is therefore a first step towards a better understanding of the evolution of micro and meso level conditions—and their interaction—over time: further research will benefit from the adoption of a similar longitudinal perspective and its application to other sectors, contexts, and forms of collaborative governance. Future efforts should also be devoted to further analyse our study’s suggestion that the combination of the three identified factors, rather than the sum of their individual effects, may play a specific role in breaking the stalemate and favouring a convergence. A more explicit configurational approach may contribute to shed light on the interacting mechanisms and dynamics that link these three factors together, thereby providing additional insights for theory and practice.

## Appendix

Appendix: ***Epilogue of the “Mose story”***

The years following the laying of the foundation stone were marked by a long implementation phase. On January 20, 2004, the Venice Safeguard Commission gave a favorable opinion on the Mose project, thus allowing the work to continue in line with the implementation plan. The Safeguard Commission, created by the Special Law for Venice^1^, was responsible for all the works carried out in the lagoon. This collegial body, where all the competent bodies and institutions are represented, is chaired by the President of the Regional Government. The favorable opinion was expressed unanimously by all fifteen members of the Safeguard Commission, among which were the representatives of the Superintendencies, the National Research Council (CNR), the UNESCO, the Ministry of Infrastructures and Transports, and that of the Agricultural Policies, the Veneto Region, the municipalities of the Venetian Lagoon, and the Venice Municipality (representative of the minority).

On February 13, 2004, the Ufficio di Piano was established by the Italian Prime Minister to ensure maximum integration between the plans drawn up by each administration responsible for safeguarding, continuity to the planned interventions, and optimal use of resources.

On May 22, 2004, the Regional Administrative Tribunal (TAR) rejected all the appeals against the Mose project lodged by some associations, including WWF and Italia Nostra, the Venice Municipality, and the Province of Venice. In 2005, after WWF and other environmental groups claimed violation of the Bird and Habitat Directives (79/409/CEE and 92/43/CEE), the European Commission initiated an infraction procedure against Italy, as it agreed that the measures to prevent deterioration of the EU protected habitats and to promote the conservation of wild birds of special protection area were not sufficient. The case was settled in 2009 after the Italian government committed to funding a plan of compensation measures and accepted that an independent party would be monitoring the works. The European Commission then proceeded to close the procedure of formal notice and additional formal notice.

However, the vicissitudes of the Mose project were not yet over. On June 4, 2014, as part of an anti-corruption investigation by the Italian judiciary, 35 people were arrested on corruption charges in connection with the Mose project. Following the judicial events between 2013 and 2014, the Italian government intervened to ensure the conclusion of the flood defense system. In December 2014, the ANAC (National Anti-Corruption Authority) proposed the extraordinary management of the consortium, followed by the appointment of three Special Chief Executive Officers. These judicial events, therefore, considerably slowed down the project.

However, this long-awaited implementation has finally come to an end: the Mose barriers were tested for the first time under effective operating conditions on October 3, 2020 and have started functioning regularly on December 31, 2021.

## References

[CR1] Adams WM, Brockington D, Dyson J, Vira B (2003). Managing tragedies: understanding conflict over common pool resources. Science.

[CR2] Ansell C, Gash A (2008). Collaborative governance in theory and practice. J Public Adm Res Theory.

[CR3] Ansell C, Gash A (2012). Stewards, mediators, and catalysts: toward a model of collaborative leadership. Innov J: Public Sect Innov J.

[CR4] Ansell C, Torfing J (Eds.) (2014) Public innovation through collaboration and design. Routledge, London

[CR5] Ansell C, Doberstein C, Henderson H, Siddiki S, ‘t Hart P (2020). Understanding inclusion in collaborative governance: a mixed methods approach. Policy Soc.

[CR6] Armitage DR, Plummer R, Berkes F (2009). Adaptive co-management for socioecological complexity. Front Ecol Environ.

[CR7] Baird J, Schultz L, Plummer R, Armitage DR, Bodin Ö (2019). Emergence of Collaborative Environmental Governance: What are the Causal Mechanisms?. Environ Manag.

[CR8] Bartelings JA, Goedee J, Raab J, Bijl R (2017). The nature of orchestrational work. Public Manag Rev.

[CR9] Beierle TA, Cayford J (2002) Democracy in practice. Resources for the Future, Washington, DC

[CR10] Biddle JC (2017). Improving the effectiveness of collaborative governance regimes: lessons from watershed partnerships.. J Water Resour Plan Manag.

[CR11] Biddle JC, Koontz TM (2014). Goal specificity: a proxy measure for improvements in environmental outcomes in collaborative governance. J Environ Manag.

[CR12] Bodin Ö (2017). Collaborative environmental governance: achieving collective action in social-ecological systems. Science.

[CR13] Bodin Ö, Mancilla García M, Robins G (2020). Reconciling conflict and cooperation in environmental governance: a social network perspective. Annu Rev Environ Resour.

[CR14] Bulkeley H, Mol AP (2003). Participation and environmental governance: consensus, ambivalence and debate. Environ Values.

[CR15] Butler JRA, Young JC, McMyn IAG, Leyshon B (2015). Evaluating adaptive co-management as conservation conflict resolution: learning from seals and salmon. J Environ Manag.

[CR16] Cheng AS, Mattor KM (2006). Why won’t they come? Stakeholder perspectives on collaborative national forest planning by participation level. Environ Manag.

[CR17] Collins BM, Stephens SL, Moghaddas JJ, Battles J (2010). Challenges and approaches in planning fuel treatments across fire-excluded forested landscapes. J Forestry.

[CR18] Cristofoli D, Trivellato B, Sancino A, Macciò L, Markovic J (2021). Public network leadership and the ties that lead. J Manag Gov.

[CR19] Cristofoli D, Douglas S, Torfing J, Trivellato B (2022). Having it all. Is it possible for collaborative governance to be both legitimate and accountable?. Public Manag Rev.

[CR20] Crosby BC, Bryson JM (2005). A leadership framework for cross-sector collaboration. Public Manag Rev.

[CR21] Crosby BC, ‘t Hart P, Torfing J (2017). Public value creation through collaborative innovation. Public Manag Rev.

[CR22] Denzin NK (1978) Sociological Methods: A Source Book. Mcgraw-Hill, New York

[CR23] Denzin NK, Lincoln YS (1994) Introduction: entering the field of qualitative research. In Denzin NK, Lincoln YS (eds) Handbook of Qualitative Research. Sage Publications, Thousand Oaks

[CR24] Douglas S, Ansell C, Parker CF, Sørensen E, ‘t Hart P, Torfing J (2020). Understanding collaboration: introducing the collaborative governance case databank. Policy Soc.

[CR25] Eisenhardt KM (1989). Building theories from case study research. Acad Manag Rev.

[CR26] Eisenhardt KM, Graebner ME (2007). Theory building from cases: opportunities and challenges. Acad Manag J.

[CR27] Emerson K, Nabatchi T, Balogh S (2012). An integrative framework for collaborative governance. J Public Adm Res Theory.

[CR28] Emerson K, Nabatchi T (2015) Collaborative governance regimes. Georgetown University Press, Washington DC

[CR29] Folke C, Carpenter S, Elmqvist T, Gunderson L, Holling CS, Walker B (2002). Resilience and sustainable development: building adaptive capacity in a world of transformations. Ambio.

[CR30] Folke C, Hahn T, Olsson P, Norberg J (2005). Adaptive governance of social-ecological systems. Annu Rev Environ Resour.

[CR31] Franklin JF, Hagmann RK, Urgenson LS (2014). Interactions between societal goals and restoration of dry forest landscapes in western North America. Landsc Ecol.

[CR32] Fliervoet JM, Geerling GW, Mostert E (2016). Analyzing collaborative governance through social network analysis: a case study of river management along the Waal River in The Netherlands. Environ Manag.

[CR33] Fullerton D (2009). Tinkering at the edges. Environ Sci Policy.

[CR34] Gray B (1989) Collaborating: Finding common ground for multi-party problems. Jossey-Bass, San Francisco, CA

[CR35] Häge FM (2013). Coalition building and consensus in the Council of the European Union. Br J Political Sci.

[CR36] Hofstad H, Torfing J (2016). Collaborative innovation as a tool for environmental, economic and social sustainability in regional governance. Scand J Public Adm.

[CR37] Huxham C, Vangen S (2005) Managing to Collaborate: the Theory and Practice of Collaborative Advantage. Routledge, London

[CR38] Imperial MT, Johnston E, Pruett-Jones M, Leong K, Thomsen J (2016). Sustaining the useful life of network governance: life cycles and developmental challenges. Front Ecol Environ.

[CR39] Innes JE, Booher DE (1999). Consensus building and complex adaptive systems: a framework for evaluating collaborative planning. J Am Plan Assoc.

[CR40] Innes JE, Connick S, Booher D (2007). Informality as a planning strategy. J Am Plan Assoc.

[CR41] Jager NW, Newig J, Challies E, Kochskämper E (2020). Pathways to implementation: evidence on how participation in environmental governance impacts on environmental outcomes. J Public Adm Res Theory.

[CR42] Locke K (2001) Grounded theory in management research. Sage Publications, London

[CR43] Margerum RD (2011) Beyond consensus: Improving collaborative planning and management. MIT Press, Cambridge

[CR44] Mariani L, Trivellato B, Martini M, Marafioti M (2022) Achieving sustainable development goals through collaborative innovation: evidence from the European Civil Society. J Business Ethic. 10.1007/s10551-022-05193-z 10.1007/s10551-022-05193-z PMC9281229 35855697

[CR45] McCaffrey S, Toman E, Stidham M, Shindler B (2013). Social science research related to wildfire management: an overview of recent findings and future research needs. Int J Wildland Fire.

[CR46] Miles MB, Huberman AM (1984) Qualitative data analysis: A sourcebook of new methods. Sage Publications, Beverly Hills

[CR47] Mostert E, Pahl-Wostl C, Rees Y, Searle B, Tabara D, Tippett J (2007). Social learning in European river-basin management: barriers and fostering mechanisms from 10 river basins. Ecol Soc.

[CR48] Newig J, Fritsch O (2009). Environmental governance: participatory, multi-level – and effective?. Environ Policy Gov.

[CR49] Ostrom E (2005) Understanding Institutional Diversity. Princeton University Press, Princeton, NJ

[CR50] Patton MQ (1990) Qualitative evaluation and research methods. Sage, Newbury Park, CA

[CR51] Pettigrew AM (1990). Longitudinal field research on change: theory and practice. Organ Sci.

[CR52] Ran B, Qi H (2019). The entangled twins: power and trust in collaborative governance. Adm Soc.

[CR53] Runhaar HAC, van Nieuwaal K (2010). Understanding the use of Science in decision- making on Cockle fisheries and gas mining in the Dutch Wadden Sea: putting the science-policy interface in a wider perspective. Environ Sci Policy.

[CR54] Ryan C (2001). Leadership in collaborative policy-making: an analysis of agency roles in regulatory negotiations. Policy Sci.

[CR55] Scott TA (2015). Does collaboration make any difference? Linking collaborative governance to environmental outcomes. J Policy Anal Manag.

[CR56] Shindler BA, Brunson MW, Stankey GH (2002) Social acceptability of forest conditions and management practices: a problem analysis. USDA Forest Service, Corvallis

[CR57] Sirianni C (2009) Investing in democracy engaging citizens in collaborative governance. Brookings Institution, Washington, DC.

[CR58] Sørensen E, Torfing J (2012). Introduction: collaborative innovation in the public sector. Innov J.

[CR59] Sørensen E, Torfing J (2021). Radical and disruptive answers to downstream problems in collaborative governance?. Public Manag Rev.

[CR60] Strauss A, Corbin J (1990) Basics of qualitative research: grounded theory procedures and techniques. Sage, Newbury Park, CA

[CR61] Stringer LC, Dougill AJ, Fraser E, Hubacek K, Prell C, Reed MS (2006). Unpacking participation in the adaptive management of social-ecological systems: a critical review. Ecol Soc.

[CR62] Susskind L, Cruikshank J (1987) Breaking the impasse: consensual approaches to resolving public disputes. Basic Books, New York.

[CR63] Torfing J (2016) Collaborative Innovation in the Public Sector. Georgetown University Press, Washington, DC

[CR64] Torfing J, Cristofoli D, Gloor P, Meier A, Trivellato B (2020). Taming the snake in paradise: combining institutional design and leadership to enhance collaborative innovation. Policy Soc.

[CR65] Trivellato B, Martini M, Cavenago D (2021). How do organizational capabilities sustain continuous innovation in a public setting?. Am Rev Public Adm.

[CR66] Ulibarri N, Emerson K, Imperial MT, Jager NW, Newig J, Weber E (2020). How does collaborative governance evolve? Insights from a medium-n case comparison. Policy Soc.

[CR67] Urgenson LS, Ryan CM, Halpern CB (2017). Visions of restoration in fire-adapted forest landscapes: lessons from the collaborative forest landscape restoration program. Environ Manag.

[CR68] Yin RK (1984) Case study research: design and methods. Sage Publications, Beverly Hills, CA

[CR69] Young JC, Marzano M, White RM, McCracken DI, Redpath SM, Carss DN, Quine CP, Watt AD (2010). The emergence of biodiversity conflicts from biodiversity impacts: Characteristics and management strategies. Biodivers Conserv.

